# In silico enhancer mining reveals SNS-032 and EHMT2 inhibitors as therapeutic candidates in high-grade serous ovarian cancer

**DOI:** 10.1038/s41416-023-02274-2

**Published:** 2023-04-29

**Authors:** Marcos Quintela, David W. James, Jetzabel Garcia, Kadie Edwards, Lavinia Margarit, Nagindra Das, Kerryn Lutchman-Singh, Amy L. Beynon, Inmaculada Rioja, Rab K. Prinjha, Nicola R. Harker, Deyarina Gonzalez, R. Steven Conlan, Lewis W. Francis

**Affiliations:** 1 Swansea University Medical School, Swansea University, Swansea, SA2 8PP UK; 2 Cwm Taf Morgannwg University Health Board, Swansea, SA2 8QA UK; 3 Swansea Bay University Health Board, Swansea, SA12 7BR UK; 4 Porvair Sciences Ltd., Wrexham, LL13 9XS UK; 5 Immunology Research Unit, GlaxoSmithKline, Medicines Research Centre, Stevenage, SG1 2NY UK

**Keywords:** Ovarian cancer, Data mining, Virtual screening, Target identification, Target validation

## Abstract

**Background:**

Epigenomic dysregulation has been linked to solid tumour malignancies, including ovarian cancers. Profiling of re-programmed enhancer locations associated with disease has the potential to improve stratification and thus therapeutic choices. Ovarian cancers are subdivided into histological subtypes that have significant molecular and clinical differences, with high-grade serous carcinoma representing the most common and aggressive subtype.

**Methods:**

We interrogated the enhancer landscape(s) of normal ovary and subtype-specific ovarian cancer states using publicly available data. With an initial focus on H3K27ac histone mark, we developed a computational pipeline to predict drug compound activity based on epigenomic stratification. Lastly, we substantiated our predictions in vitro using patient-derived clinical samples and cell lines.

**Results:**

Using our in silico approach, we highlighted recurrent and privative enhancer landscapes and identified the differential enrichment of a total of 164 transcription factors involved in 201 protein complexes across the subtypes. We pinpointed SNS-032 and EHMT2 inhibitors BIX-01294 and UNC0646 as therapeutic candidates in high-grade serous carcinoma, as well as probed the efficacy of specific inhibitors in vitro.

**Conclusion:**

Here, we report the first attempt to exploit ovarian cancer epigenomic landscapes for drug discovery. This computational pipeline holds enormous potential for translating epigenomic profiling into therapeutic leads.

## Introduction

The last decades have witnessed a significant increase in functional genomic approaches, high-throughput data availability and innovative computational methodologies [1]. Thriving in this data-intensive environment, in silico strategies have been widely used for drug development, discovery and repositioning [2]. In silico approaches take advantage of vast genomic datasets to stratify patients into distinguishable molecular sub-groups, enabling the delivery of more accurate personalised care to patients [3, 4]. To date, this field has largely been driven by genomics, whereas now there is a surge in the exploitation of epigenomics, which offers a more dynamic view of disease, and therefore personalised treatment options [5, 6].

In contrast to genomics, epigenomics encompasses a set of covalent modifications that occur at chromatin level with no alteration to the underlying DNA sequence [7]. Different patterns of epigenetic alterations have fundamental effects on cancer progression, providing cancer cells with a degree of plasticity that allows them to adapt to their environment [8]. The precise temporal and spatial epigenomic regulation of gene transcription is orchestrated by cis-acting regulatory elements that include promoter regions surrounding the transcriptional start site of genes, and enhancers, generally located many kilobases away from the gene body [9]. Active enhancers are highlighted by acetylated histone 3 at lysine 27 (H3K27ac), an epigenetic mark commonly associated with open chromatin regions [10]. Enhancers play a central role in regulating transcription where disease-specific alterations are a critical aspect of cancer development, as aberrant enhancer landscapes alter the expression of oncogenes and tumour suppressor genes [11]. Re-programmed enhancer landscapes have been identified in several cancers, including ovarian cancer (OC) [12, 13].

OC malignancies are subdivided into histological subtypes that exhibit distinct gene expression profiles, clinical features, response to chemotherapy and outcome [14]. High-grade serous carcinomas (HGSC) are the most common (~75%) and most aggressive subtype, while other histological presentations are much less prevalent, for example clear-cell (CC, ~5%) and mucinous carcinomas (MC, 2–3%) [14]. Despite OC heterogeneity, the first line of standard treatment is generally comprised of debulking surgery followed by platinum-based chemotherapy [15]. Although most cases initially display a positive response to this treatment strategy, 40–50% patients develop platinum resistance, leading to an unfavourable prognosis [16]. At present, only a few targeted therapies have been approved for use in the management of OC and include PARP and VEGF inhibitors, however specific resistance mechanisms have also emerged against these treatment strategies [17, 18].

Here, we present a novel computational strategy designed to identify potential and much-needed candidate drugs for OC treatment. Interrogating publicly available epigenomic ChIP-Seq data, this pipeline mapped differential enhancer regions between diseased and healthy samples. Using these regions as input for the ‘Mining Algorithm for Genetic Controllers’ algorithm (MAGIC) [19], specific transcription factor (TF) binding enrichment was determined. Finally, candidate drug compounds were identified using the ‘Search Tool for Interactions of Chemicals’ (STITCH) [20], and experimental validation was conducted using patient-derived OC samples. Among the predicted top-ranked candidates specific for HGSC we identified CDK inhibitor SNS-032 and EHMT2 inhibitor UNC0646, both of which exhibited promising in vitro results. Importantly, our novel framework can now be exploited in multiple disease settings, offering a unique approach to drug discovery through profiling of epigenomic landscapes.

## Methods

### Dataset download and quality control assessment

ChIP-Seq datasets were downloaded from repositories GSE68104, GSE121103 and GSE156275 [12, 21–23]. The sample size was determined by public availability. Fastq files were downloaded using the prefetch and fasterq-dump functions from the SRA toolkit. Prior to analysis, the quality of raw data was assessed using fastqc [24]. Read mapping statistics were obtained directly from Bowtie2 and STAR aligners for ChIP-Seq and RNA-Seq respectively [25, 26]. For ChIP-Seq samples (H3K27ac), read quality was assessed using the phantompeakqualtools package [27]. Normalised strand cross-correlation and relative strand cross-correlation metrics were required to adhere to the ENCODE standards [28].

### Next-generation sequencing analysis

For ChIP-Seq analysis, sequence reads were mapped against human reference genome build hg38 using Bowtie2 [25]. Removed reads prior to peak calling include: reads >5 bases with Phred score <30, reads containing undefined bases and reads mapping to DAC ENCODE consensus excluded regions [29]. Peak calling was performed using MACS2 with default settings [30]. To identify significantly dysregulated H3K27ac peaks, we used the Bioconductor package DiffBind with DESeq2 settings [31, 32]. DiffBind was used to analyse ChIP-Seq signal distribution in genomic locations of annotated enhancers obtained from the GeneHancer database [33], incorporating the remove grey list and remove blacklist options. Enhancer occupancy heat maps were generated using the pheatmap R package [34]. Principal component analysis was performed and plotted using DiffBind [35] and ggplot2 [36] packages, respectively. Volcano plots and pathway enrichment plots were also generated using ggplot2. Genome browser plots were generated using Gviz R package [37]. Upset plots were generated using UpSetR package [38]. Venn diagrams were created using Inkscape v0.92 [39]. Pathway analyses were performed using WebGestaltR [40]. For RNA-Seq analysis, sequence reads were mapped against human reference genome build hg38 using STAR [26]. Prior to alignment, low-quality read ends were removed using the sickle window adaptive trimming tool [41]. Gene count tables were generated using the STAR aligner quant mode function.

### MAGIC enhancer matrix and transcription factor enrichment

As MAGIC was originally designed to assess enriched TF binding in gene promoter regions, a novel MAGIC matrix was designed to map enhancer locations annotated in the GeneHancer database [33]. We developed a custom R script to generate our matrix, where 1695 BED files from TF ChIP-Seq experiments for both human cell lines and primary samples were downloaded from ENCODE using the ENCODExplorer R package (R version 3.6.3) [42]. With this newly generated matrix, MAGIC was used to identify enriched TF binding within enhancers (FDR < 0.01, K-S test). ‘Find Individual Motif Occurrences’ (FIMO) [43] was used to refine the list of TFs produced by MAGIC, removing TFs with no significant enrichment of motif binding sites (*P* < 0.001) using a custom R script. Motif sequence logos were generated using the diffLogo R package [44]. Molecular surface render plots of complex members were obtained from Uniprot [45] and UCSF Chimera [46].

### Protein complex and compound search

The CORUM DB protein complex database [47] was used to isolate enriched protein components using a custom R script. The STITCH database was used to assess chemicals linked to specific complex coregulators, filtered and ranked using a STITCH combined score (>700) [20]. The ChEMBL database was used to identify chemicals corresponding to drug compounds approved by the FDA or undergoing clinical trial [48]. Bar plots, RPKM complex factor diagrams and pie charts were generated using ggplot2 [36]. Network diagrams were generated using Cytoscape [49].

### Compounds, cell culture and cell viability assays

Identified drug compounds were provided by GlaxoSmithKline (i-CBP112, GSK343, GSK503, UNC0646), purchased from Cayman Chemical (MI, US): Seliciclib (CAY10009569), BIX-01294 (CAY13124), SNS-032 (CAY17904) and NU-6102 (CAY113317) or MedChemExpress (NJ, US): AUZ 454 (HY-15004). Patient-derived primary ovarian cancer cells were isolated from ovarian biopsies using a protocol adapted from Sheperd et al. [50]. Human biological samples were sourced ethically and their research use was in accord with the terms of the informed consents under an IRB/EC-approved protocol. Primary cells were maintained in 50% MCDB 105 + 50% M199 media (Merck, DE) supplemented with 20% foetal calf serum (ThermoFisher Scientific, MA, USA). Sample size was determined by availability. Ovarian cancer cell lines were purchased from ATCC^®^ (LGC Ltd, UK). SKOV3 (CVCL_0532) and OVCAR-3 (CVCL_0465) were maintained in RPMI 1640 media (Gibco^™^, 11875093) supplemented with 20% foetal calf serum (FBS; Gibco^™^, 10270106) and 10 μg/ml of insulin solution from bovine pancreas (I0516, Merck; DE). UWB1.289 (CVCL_B079) and CAOV3 (CVCL_0201) were maintained in DMEM/F-12+GlutaMAX^™^ (ThermoFisher Scientific) (10% FBS). All cell lines used within this study were authenticated at NorthGene^™^ using the PowerPlex^®^ Fusion kit (DC24, Promega; WI, USA) in 2022. Laboratory reports are available upon request. Micoplasma contamination was routinely tested with the MycoAlert Mycoplasma Detection Kit (LZLT07218, Lonza, CH). The RealTime-Glo^™^ MT Cell Viability Assay (Promega, G9712) was used to assess live cell viability following the manufacturer’s instructions. All samples were tested in triplicates (*n* = 3). The experiment was replicated three times in the laboratory. Half-inhibitory concentration values (IC_50_) and heatmaps were generated using Graphpad Prism (V9). Raw luminescence values were transformed into logarithmic values, normalised and fitted to a dose-response curve using a non-linear regression; 100% and 0% values were defined by vehicle control and Staurosporine positive control (Biotechne, MN, USA), respectively.

### Flow cytometry

All flow cytometry experiments were undertaken using a CellStream^®^ (Luminex; TX, US). Cell cycle staining was performed using DAPI ready-made solution (1 µl/mL, MBD0015; Merck, DE) following the manufacturer’s instructions. Prior to staining, cells were fixed in 70% ethanol. Cell cycle profiles were analysed using FlowJo^™^ 10 using the Watson (Pragmatic) model; all data met the assumptions of the test. Schematic representations of cell percentages in different cell cycle phases were graphed using Graphpad Prism (V9). Values shown are defined as mean; error bars represent the coefficient of variation. Apoptosis staining was performed using Annexin V-FITC (640906, Biolegend; CA, US) and Propidium iodide (PI, 421301, Biolegend) as per manufacturer’s instructions, which included the use of Annexin V Binding Buffer (422201, Biolegend) and Cell Staining Buffer (420201, Biolegend). Unstained cells and cells stained with Annexin V/PI only were used to calculate the compensation matrix that was applied to all the data to adjust for signal overlap between channels of the emission spectra. All data met the assumptions of the test.

## Results

### Epigenomic landscapes reveal novel therapeutic avenues

**Fig. 1:**
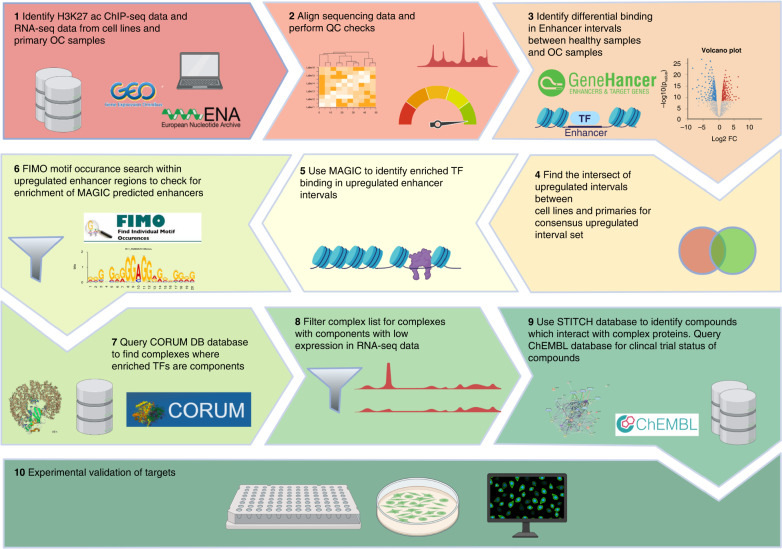
Schematic diagram of the computational pipeline approach. The diagram was created with BioRender.com.

Deep epigenomic data mining offers an exciting alternative approach to current computational drug repurposing pipelines that typically use genomic or transcriptomic datasets [51, 52]. In this study, we implemented a novel computational approach to identify potential candidates for the treatment of OC subtypes (Fig. 1) using both cell line and primary sample H3K27ac ChIP-Seq datasets as discovery cohorts. Specifically, five primary OC sample datasets per subtype (HGSC, CC, MC 1–5) and three HGSC (Kuramochi, OAW42, UWB1.289), CC (JHOC5, RMG-II, ES2) and MC (MCAS, GTFR230, EFO27) cell line datasets were explored (Supplementary Table S1). Datasets from patient-derived primary ovarian samples (IOE4 and IOE11) were used as non-cancerous controls [21].

### Prediction of regulatory elements in ovarian cancer ChIP samples

**Fig. 2:**
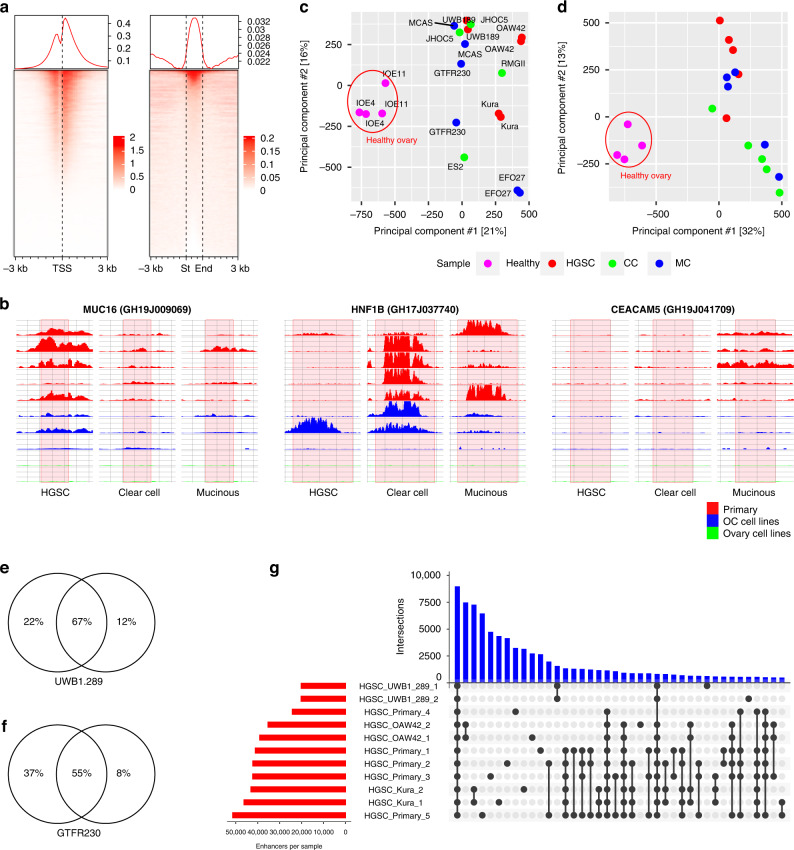
Basal analysis of H3K27ac signal in ovarian cancer subtypes. **a** Genome wide coverage of H3K27ac ChIP signal around promoter and enhancer sites in HGSC primary samples. **b** Counts per million of H3K27ac ChIP signal in GH19J009069 MUC16 interacting enhancer, HNF1B GH17J0377409 interacting enhancer and GH19J041709 CEACAM5 interacting enhancer in representative HGSC, CC and MC primary cells (red), cell lines (blue) and healthy samples (green). Red highlighted areas indicate the extent of enhancer regions. **c**, **d** Principal component analysis of ChIP signal in GeneHancer enhancer sites of healthy cells and ovarian cancer cell lines or primary cells. **e**, **f** Overlaps of H3K27ac peaks in replicates of UWB1.289 and GTFR230 cells. **g** Intersections of H3K27ac occupied GeneHancer enhancer regions for all HGSC samples analysed. See also Supplementary Fig. S1 for intersections of CC and MC samples.

Extensive quality control was performed on NGS data as samples were derived from different sources (Supplementary Table S1). Based on standard quality check parameters [53], all samples were deemed acceptable to pursue further analysis. At first, we undertook peak calling using MACS2, which resulted in the identification of an average of 65,000 peaks per sample. To facilitate downstream analyses with MAGIC [19], enriched regions were also identified using annotated enhancers obtained from the GeneHancer database [33]. The GeneHancer repository contains ~400,000 human regulatory elements divided into proximal (promoter), distal (enhancer) and proximal with distal enhancer functions. Using this approach, we identified 11,485–70,139 predicted regulatory elements per sample (Supplementary Table S1). Signal enrichment of proximal elements displayed a bimodal genomic distribution, while distal and proximal/distal elements displayed a unimodal genomic distribution (Fig. 2a), as expected [54, 55]. Due to H3K27ac being considered primarily as an active enhancer mark [10], we decided to concentrate on the examination of distal and proximal/distal enhancer elements, henceforth simply labelled as enhancers.

To validate predicted enhancer locations at genomic level, we evaluated specific enrichment at the sites of well-known biomarkers of OC. MUC16 is the most widely used biomarker for OC [56] and we found enhancers linked with its expression in all HGSC samples and 50% of CC (Fig. 2b). HNF1B is commonly regarded as a CC-specific biomarker [57]. In accordance, an enhancer located proximally to its transcriptional start site was enriched in most CC samples but not in HGSC (Fig. 2b). CEACAM5 is a known tumour marker specific for MC [58] and analysis identified that corresponding enhancers were enriched in most MC samples (Fig. 2b).

Prior to differential analysis, we evaluated broad enhancer landscape similarities and differences, and discovered a clear contrast between cell line profiles of diseased and healthy samples (Fig. 2c). Separate clustering was also observed when analysing profiles of primary cells of all subtypes (Fig. 2d). In most instances replicates clustered together, confirming the high reproducibility of samples (Fig. 2c–f). To compare the persistence of enhancer regions across subtypes, we searched for MACS2 peaks which overlapped with enhancer regions in the GeneHancer database. Comparison of overlapping regions in HGSC revealed a total of 112,256 enriched enhancers across all samples, of which 12.8% were present in all primary samples and 9.6% were present in all cell line samples (Fig. 2g). In the case of CC (6.8% primaries, 15.5% cell lines) and MC (9.5% primaries, 20.5% cell lines), the overlap is higher in cell lines compared to primary samples (Supplementary Fig. S1). The largest intersection of enhancers occurred when comparing all primary samples and cell lines, suggesting a recurrent but still highly privative and sample-dependent enhancer landscape, as has been noted in cancer cells of colon, gastric and ovarian origin [59–61].

### Differential analysis reveals subtype-specific target enhancer landscapes

**Fig. 3:**
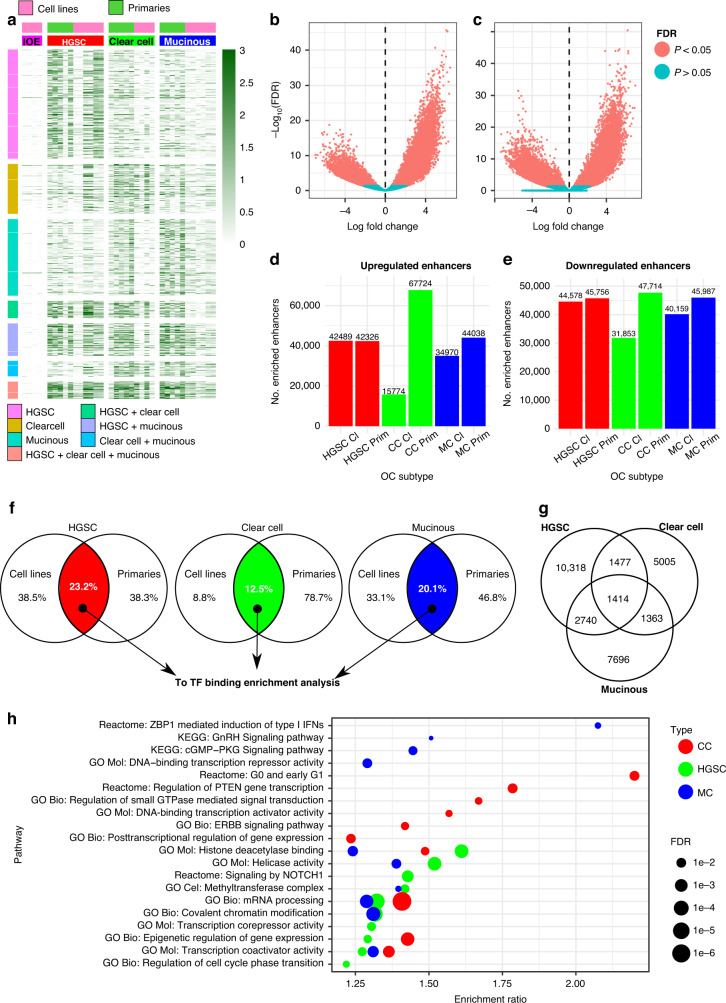
Differential analysis of H3K27ac signal between cancerous and healthy samples. **a** Reads per kilobase of transcript, per million mapped reads (RPKM) normalised H3K27ac ChIP signal for GeneHancer enhancers with significantly enriched H3K27ac binding in OC subtypes. **b**, **c** Volcano plots showing dysregulation of H3K27ac ChIP signal in healthy cells versus HGSC cell lines and HGSC primary samples. **d**, **e** Number of enriched and under-represented GeneHancer enhancer regions in HGSC, CC and MC cell line and primary samples extracted from the differential analysis results (FDR < 0.05). **f** Intersection of enriched GeneHancer enhancer sites in cell line and primary samples for all HGSC, CC and MC samples. **g** Intersection of final lists of enriched enhancers for all HGSC, CC and MC samples. **h** Examples of significantly enriched pathways for genes associated with specific GeneHancer enhancers in OC samples.

Next, we performed differential analysis comparing the peak strength of H3K27ac-enriched regions between cancerous and healthy samples (Fig. 3a). We identified a total of 88,245 enhancer regions that were significantly enriched across the three HGSC cell lines (Fig. 3b), whereas 86,904 were identified when comparing HGSC primary cells and healthy samples (Fig. 3c). Differential analysis of CC and MC cell lines and primary samples rendered analogous peak representation, while primary enriched peaks were significantly greater than active enhancers in cell line samples (Fig. 3d, e). Given that enhancer enrichment, as determined by elevated levels of H3K27ac, signifies an open chromatin architecture, we were keen to understand what this effect could have on TF and concomitant coregulator recruitment. We therefore focused only on those enhancer regions that were enriched, and common to both the cell line and primary sample datasets.

In the case of HGSC, we found an overlap of 23.2% between cell lines and primary samples, while we observed 12.5% and 20.1% in CC and MC, respectively (Fig. 3f). Interestingly, we found a global overlap of 4.7% between the intersected enriched enhancer regions of the different subtypes. A relatively high percentage of cell type-specific enhancers were also observed in HGSC (34.3%), CC (16.7%) and MC (25.6%) (Fig. 3g). Lastly, we assigned enriched enhancer locations to putative target genes based on GeneHancer annotations and performed pathway analyses using WebGestaltR [40]. Subtype-specific regulatory networks were found to be strongly associated with predicted enriched enhancer regions (FDR < 0.05), including NOTCH1 signalling pathways, known to be altered in HGSC [62] and the ERBB signalling pathway, linked with CC [57] (Fig. 3h). These results suggest that there is a predominance of subtype-specific enhancer landscapes, corroborating earlier observations [12, 13, 61].

### Identification of enriched transcription factor binding

**Fig. 4:**
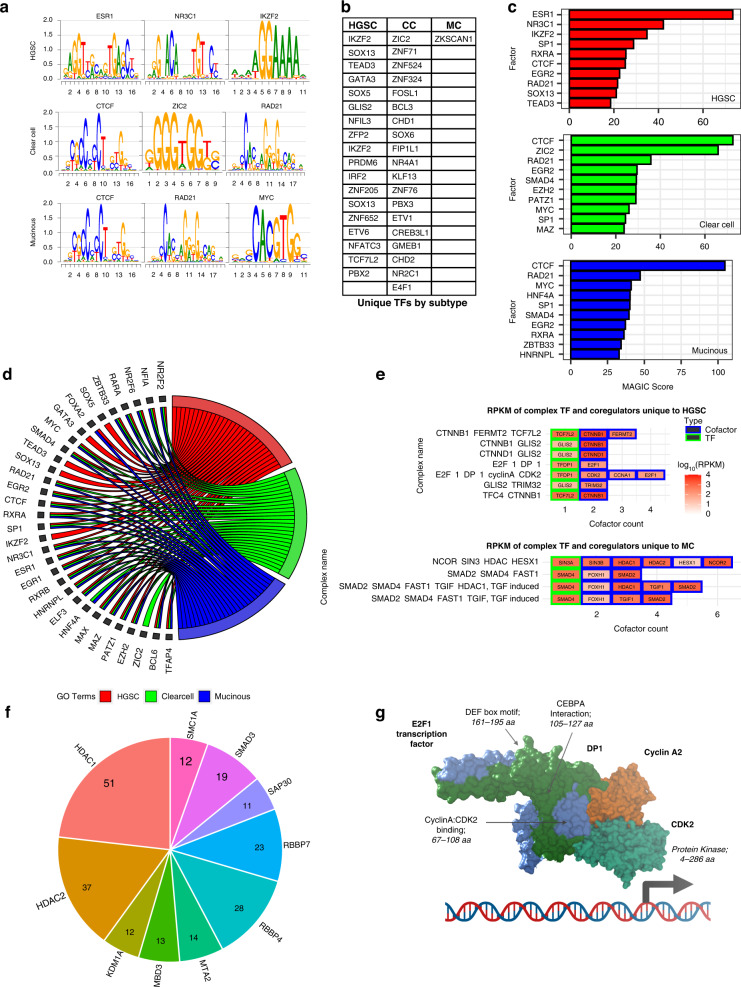
Identification of enriched transcription factor binding and associated complexes. **a** Binding motifs identified by FIMO for top three predicted enriched TFs in enriched HGSC, CC and MC enhancer sites. **b** Subtype unique TFs with enriched binding in enriched enhancer sites. **c** MAGIC score of top ten TF binding sites for enriched GeneHancer enhancer sites in HGSC, CC and MC subtypes after filtering using MOTIF analysis and RNA-Seq expression levels. **d** Subtype-specific significant enrichment of TFs for the top twenty significantly enriched TFs from each subtype. **e** RPKM score from RNA-Seq HGSC and MC primaries for complexes only identified in HGSC and MC samples, respectively. Green borders indicate TFs identified in the pipeline and blue borders indicate complex coregulators. **f** Number of complexes where each coregulator is a component for HGSC samples, including only coregulators present in more than ten complexes. See also Supplementary Fig. S2 for coregulators of CC and MC samples. **g** E2F-1-DP-1-Cyclin A-CDK2 complex identified uniquely in HGSC. Created with BioRender.com.

Subsets of aberrantly over-represented H3K27ac peaks were analysed using MAGIC and our custom enhancer matrix, with 534 TFs exhibiting significant enrichment (*P* < 0.001). This list was cross-referenced with publicly available RNA-Seq data to filter out non-expressed TFs (GSE121103) [12], resulting in the identification of 164 TFs that displayed significant enrichment scores across the OC subtypes (Fig. 4a and Supplementary Table S2). Of these, 100 (61%) were shared amongst all subtypes, 28 between two subtypes (17%), and 36 (22%) were subtype-specific (Fig. 4b). ESR1 and NR3C1were the predominant TFs (Fig. 4c, d), both of which have been previously associated with ovarian carcinogenesis [63, 64]. ESR1 was also the top enriched TF for the HGSC subtype (Fig. 4c), suggesting a major role for this TF in regulating aberrant epigenomic landscapes that lead to HGSC tumour development. As well as hormone receptors, we identified other major general TFs, like MYC (Fig. 4c, d), a TF that has been associated with all stages and types of ovarian tumorigenesis [65].

### Identification of enriched complex coregulators

Transcriptional regulation involves the recruitment of dynamic complexes to regulatory regions either subsequently to or together with the direct binding of TFs [66]. Accordingly, we interrogated the CORUM database to identify coregulators that are known to associate with each of the TFs linked with the deregulation of OC enhancer landscapes [47]. After removing non-expressed coregulators using RNA-Seq data, analysis resulted in the identification of 201 regulatory complexes, of which 175 (87%) were shared amongst all subtypes, 15 between two subtypes (7.5%), and interestingly 11 (5.5%) were subtype-specific for either HGSC or MC, suggesting that OC subtype-specific regulatory complexes could exist (Fig. 4e and Supplementary Table S3). HGSC subtype-specific complexes include E2F-1-DP-1-cyclin A-CDK2 and CTNNB1-FERMT2-TCF7L2, while MC-specific complexes are mainly formed by interacting SMAD coregulators (Fig. 4e–g and Supplementary Table S3). Many of the coregulators identified are known to be present in several of the 201 protein complexes and were extensively shared amongst the subtypes (259 out of 290), while only a small percentage were privative of specific subtypes (8 HGSC, 4 CC and 19 shared between 2 different subtypes) (Fig. 4e and Supplementary Table S3). Amongst the more prevalent cofactors, HDAC1 and HDAC2 were present in approximately 20-30% of the identified complexes across OC subtypes (Fig. 4f and Supplementary Fig. S2).

### Coregulator interactions reveal potential drug candidates

Having identified TFs and coregulators associated with aberrant enhancer switching, we next sought to identify chemical compounds that could be used to target these regulatory proteins by assigning chemical/protein interaction scores via STITCH [20]. Despite differences in subtype-specific enhancer landscapes, potential drug candidates were largely shared between subtypes, with an 87.9% overlap likely due to shared TFs and coregulators. The top ten ranked drug candidates for all subtypes were monopolised by those directly targeting ESR1 and NR3C1 (Table 1 and Supplementary Table S4). Interestingly, Sulindac, a non-steroidal anti-inflammatory identified in previous in silico repositioning studies [51], was ranked among the top compounds for all subtypes (Table 1).

**Table 1:** Top-ranked compounds predicted to target OC cells based on subtype-specific enhancer landscapes.

OC subtype	Target	Drugs	Comments
High-grade serous, clear cell and mucinous carcinomas	PPARG	Rosiglitazone, Pioglitazone Telmisartan, Rapamycin	Anti-diabetic and anti-inflammatory drugs. First two withdrawn from market in several countries
	ESR1	Fulvestrant, estradiol, tamoxifen, afimoxifene	Oestrogen receptor modulators
	NR3C1	Mifepristone, hydrocortisone, dexamethasone	Anti-glucocortidoids and glucococorticoid medication
	CTNNB1	Sulindac, dinoprostone, indomethacin	Non-steroidal anti-inflammatories (NSAIDs)
High-grade serous carcinomas	CDK2	Seliciclib, SNS-032	Tested in vitro
	EHMT2	Flavopiridol, BIX-01294, UNC0646	Tested in vitro
	EZH2	Ademetionine, GSK503, GSK343	Tested in vitro
Clear-cell carcinomas	SRC, CDK7	Dasatinib, Alvocidib	—
Mucinous carcinomas	NFYA, FOXH1	Icosapent, Galzin	—

**Fig. 5:**
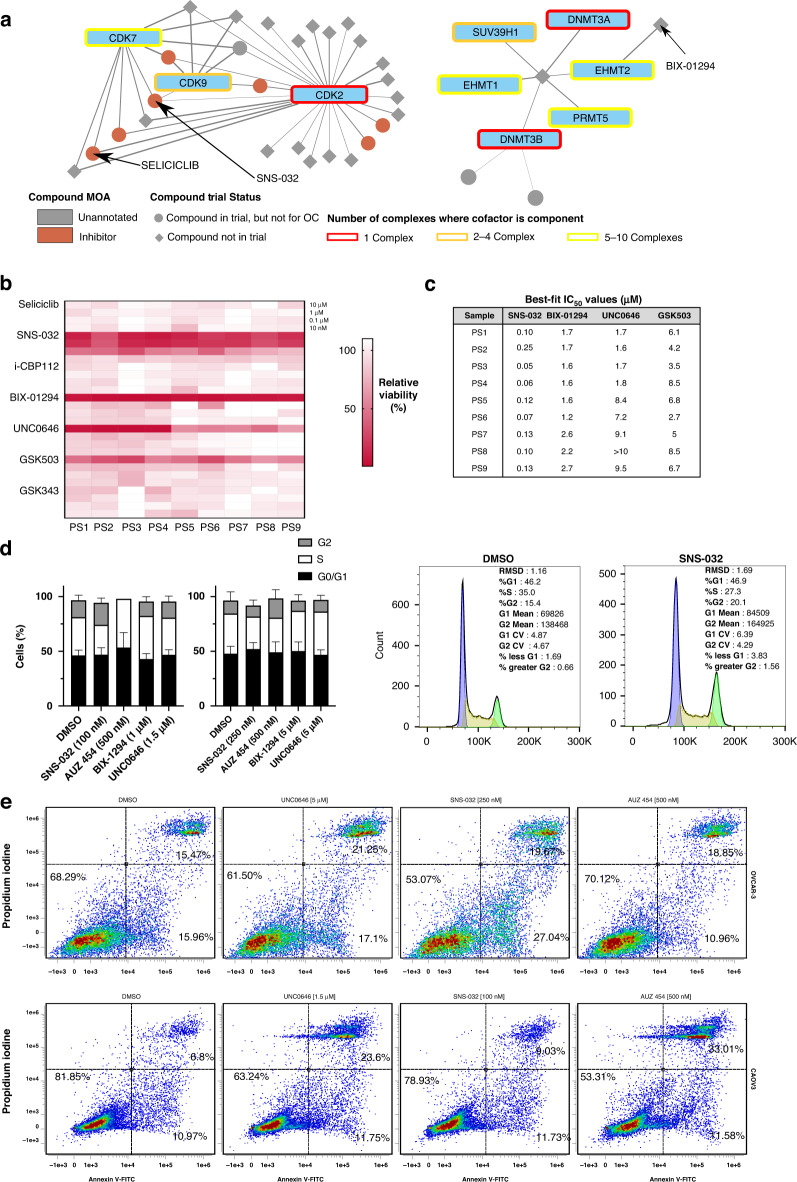
Drug compound selection and validation of drug efficacy using clinical samples. **a** Network diagram of HGSC targets (CDKs and EHMT2) and targeting compounds (STITCH score >0.95). Edge thickness represents combined STITCH confidence score. Round nodes and diamond nodes represent compounds that have undergone some phase of clinical trials and have not undergone clinical trials, respectively. Compound node colours represent compound mechanism of action (MOA). Factor node outline colour indicates the number of the expressed complexes which the factor is a component of. Red and green highlight circles indicate CDK and EHMT-related protein groups respectively. **b** Heatmap representing the effect of drug compounds on patient-derived clinical samples treated for 72 h. Each 4 rows of the heatmap represent the top concentrations used to derive IC_50_ values (10 μM, 1 μM, 100 nM and 10 nM). Heatmap values were calculated using relative viability compared to the vehicle control (DMSO). Red colour indicates low viability following treatment. **c** Table displaying calculated best fit IC_50_ values after 72 h of treatment with varying concentrations (10 pM-10 μM) of displayed drug compounds. DMSO was used as vehicle control and staurosporine was used as positive control (+). **d** Flow cytometry cell cycle analyses of CAOV3 and OVCAR-3 cells treated with selected compounds for 24 h. Bar chart error represents coefficients of variation (cv). Blue peaks represent cells in G0/G1, while green peaks represent cells in G2/M phase. The area depicted as yellow represents cells in the S phase. **e** Flow cytometry apoptosis analysis of CAOV3 and OVCAR-3 cells treated with selected compounds for 24 h. Cells were stained with Propidium iodide and Annexin V-FITC, rendering 4 populations: viable (−, −), early apoptotic (−,+), late apoptotic (+, +) and dead (+, −), three of which are highlighted in the panels. Graphs display cell densities, whereby red, green and blue colours indicate high, medium and low cell densities, respectively.

Screening identified seliciclib/CYC202 and SNS-032/BMS-387032 as HGSC-specific molecules that inhibit CDK2, 7 and 9 [67, 68] (Fig. 5a and Table 1). Both drug compounds have been investigated for the treatment of solid malignancies in Phase I clinical trials [69, 70] but they did not progress to Phase II. In addition, we broadened for potential therapeutic candidates search, to identify drugs that have not yet been included in clinical trials for any cancer setting by exploring the CHEMBL database [48] (Supplementary Table S4). Using this approach we identified BIX-01294, a molecule that inhibits histone methyltransferase EHMT2/G9A, involved in anti-tumour immune response and chemoresistance in HGSC [71] (Fig. 5a and Table 1).

### Validation of predicted HGSC-specific therapeutics

The efficacy of selected molecules was evaluated in primary cells derived from late-stage HGSC clinical samples and cell line models (Fig. 5b, Supplementary Table S5 and Supplementary Fig. S3). The histone acetyltransferase inhibitor i-CBP112 ranked poorly (~400th) and was selected as a negative control. Overall, SNS-032 exhibited potent and clinically achievable anti-tumour activity across the panel of patient samples and cell lines; seliciclib and i-CBP112 showed little to no activity (IC_50_ > 10 μM) (Fig. 5b, c and Supplementary Fig. S3). Although SNS-032 was primarily selected based on its activity against CDK2, it is also sensitive to CDK7/9 (IC_50_ in cell-free assays: 4 nM [CDK9], 48 nM [CDK2] and 62 nM [CDK7]) [67]. To validate the specific vulnerability of HGSC towards CDK2 inhibition, we tested the CDK1/2 inhibitor NU-6102 and the CDK2-specific inhibitor AUZ 454. Whilst NU-6102 showed no activity (IC_50_ > 10 μM), probably due to its higher sensitivity towards CDK1, AUZ 454 exhibited considerable levels of anti-tumour activity in the range of μM (Supplementary Fig. S3), suggesting an abnormal behaviour of CDK2 in HGSC. Next, we performed qRT-PCR and western blot analyses to confirm predicted modes of action. The expression of known CDK2 targets was significantly upregulated following SNS-032 treatment (Supplementary Fig. S4) [72]. At the same time, CDK2-specific target Rb-Thr821 was de-phosphorylated after exposure with SNS-032 and AUZ 454 [73], indicating the presence of a CDK2-specific mechanism of action. In addition, we performed cell cycle and apoptosis assays which, as expected, led to apoptotic cell death coupled with cell cycle arrest (Fig. 5d, e and Supplementary Fig. S5).

We also tested compounds BIX-01294 and UNC0646 targeting EHMT2 as well as GSK503 and GSK343 targeting methyltransferase EZH2 as means of comparison. After 72 h, both EHMT2 inhibitors exhibited potent anti-tumour in vitro activity in all models (Fig. 5b, c and Supplementary Fig. S3). Neither of the EZH2 inhibitors was able to match the in vitro efficacy observed with BIX-01294 or UNC0646 (Fig. 5b, c and Supplementary Fig. S3). The expression of known EHMT2 targets was significantly up and/or downregulated following treatment [74], and EHMT2 inhibition led to a significant decrease in the levels of H3K9me2, the main histone methylation target of EHMT2 (Supplementary Fig. S4); these results point to a specific EHMT2-driven effect. EHMT2 inhibition led to increased levels of cellular apoptosis, whereas no significant changes were observed in the DNA profile (Fig. 5d, e and Supplementary Fig. S5). Overall, the results of in vitro validation studies in patient-derived cells and cell lines further confirmed the efficacy of our approach, supporting the concept of epigenomic profiling as means to guide therapeutic strategies.

## Discussion

In 2012, the ENCODE project proposed that there were 400,000 regions with enhancer-like features in the human genome [75]. Since then, numerous studies have investigated the epigenomic landscape of cancer, labelling active enhancers in the regions of tens of thousands per cell type. For example, Lidschreiber et al identified 58,457 putative enhancers across 7 cancer cell lines from different origins [76], Mack et al. profiled more than 30,000 enhancers in ependymoma primary cells [77] and Ooi et al. detected 36,973 predicted distal enhancer regions in gastric cancer cell lines [60]. In ovarian cancer, Shang et al identified 36,388 regulatory elements across four cell line models [61]. We have refined this approach to define cancer-associated enhancer landscapes and have identified aberrant enhancer activity associated exclusively to specific OC subtypes. Linking broad epigenomic signatures, in this case H3K27ac, we were subsequently able to map enhancer locations to enriched TF binding sites and associated coregulator proteins. Interestingly, through this process, we observed a notable disparity between the subtype-specific nature of most abnormal cis-regulatory elements and the fact that the TFs, and more pronouncedly, the cofactors governing such networks display substantial homogeneity across the range of studied OC subtypes.

To discover novel therapeutic agents for OC, we sought to identify chemicals that targeted the TFs and coregulators uniquely enriched at enhancers. To validate our findings and the usefulness of our approach, we tested the in vitro efficacy of a subset of pipeline drug outputs in patient-derived primary samples. SNS-032 is a potent and selective CDK2, 7 and 9 inhibitor [67], whose in vitro efficacy has been proven in OC cell lines, and their sensitivity towards this compound linked with cyclin E1 overexpression, relatively common in HGSC patients (15–20%) [78]. SNS-032 was investigated for the treatment of haematologic and solid malignancies in two Phase I clinical trials [69, 70] but no further developments have been undertaken. We extended previous investigations on the effect of SNS-032 in OC using patient-derived primary samples and cell lines, although unfortunately, we could not confirm sample CCNE1 status. Our study suggests that SNS-032 represents a good therapeutic option for HGSC patients, given its outstanding efficacy in vitro. In contraposition, seliciclib or NU-6102 displayed overall lower in vitro efficacy levels, which correlates with known IC_50_ values obtained in cell-free assays as well as previous in vitro observations [79]. Importantly, CDK2 was one of the few cofactors uniquely identified in HGSC, as part of the protein complex E2F-1-DP-1-cyclin A-CDK2 [80]. Our findings highlight the critical role of this cofactor modulating broad epigenomic landscapes and demonstrate a potential for further research into the clinical effectiveness of targeting CDK2 in HGSC.

In our effort to put forward new therapeutic targets against HGSC, we performed further in vitro testing of compounds targeting EZH2 and EHMT2 molecules. Preclinical studies have previously highlighted the potential of targeting EZH2, although no clinical trial is currently investigating the use of EZH2 inhibitors, specifically in OC subjects [81]. The EHMT1/2 inhibitor BIX-01294 is known to block the proliferation of cancer cell lines but has shown considerable toxicity problems in preclinical studies [82]. We have shown that while EZH2 inhibitors mainly displayed poor in vitro efficacy results, BIX-01294 analogue UNC0646 exhibited an improved response, with IC_50_ values in the range of ~1–3 μM across the studied samples.

Epigenomic deregulation continues to emerge as a key driver in ovarian tumorigenesis, and more specifically in the differentiation of OC subtypes [23]. Such epigenomic deficiencies have the potential to be targeted with existing therapies already in clinical or preclinical trials for other diseases. Here we present the first attempt to investigate subtype-specific enhancer re-programming aimed at drug discovery and repurposing in OC. Overall, in silico and in vitro investigations suggest that EHMT2, together with CDK2, represent the best candidates to pursue further preclinical studies aimed at improving HGSC treatment strategies. The effectiveness of this approach, identifying potential candidate molecules based on the evaluation of one histone mark alone, is extremely encouraging. Combining enhancer-localised epigenomic datasets for H3K27ac and other histone marks could lead to further subtype stratification using this approach.

## Supplementary information

Supplementary Information

Supplementary Table S1

Supplementary Table S2

Supplementary Table S3

Supplementary Table S4

## Data Availability

The datasets generated during and/or analysed during the current study are available from the corresponding author on reasonable request.

## Code availability

Code and usage instructions are available at https://github.com/RBGO-Lab/EnhancerDrugTargetPipeline.
