# Supplementary material for: In silico enhancer mining reveals SNS-032 and EHMT2 inhibitors as therapeutic candidates in high-grade serous ovarian cancer
Source: Br J Cancer. 2023 Apr 29;129(1):163–74. doi: 10.1038/s41416-023-02274-2 (PMC10307814; doi:10.1038/s41416-023-02274-2)
Supplement: Supplementary file 1 — Supplementary Information [file 41416_2023_2274_MOESM1_ESM.docx]

**Supplementary Figures**

**Supplementary Figure S1.** Intersection of MACS2 peaks with GeneHancer enhancers in clear cell and mucinous carcinoma samples


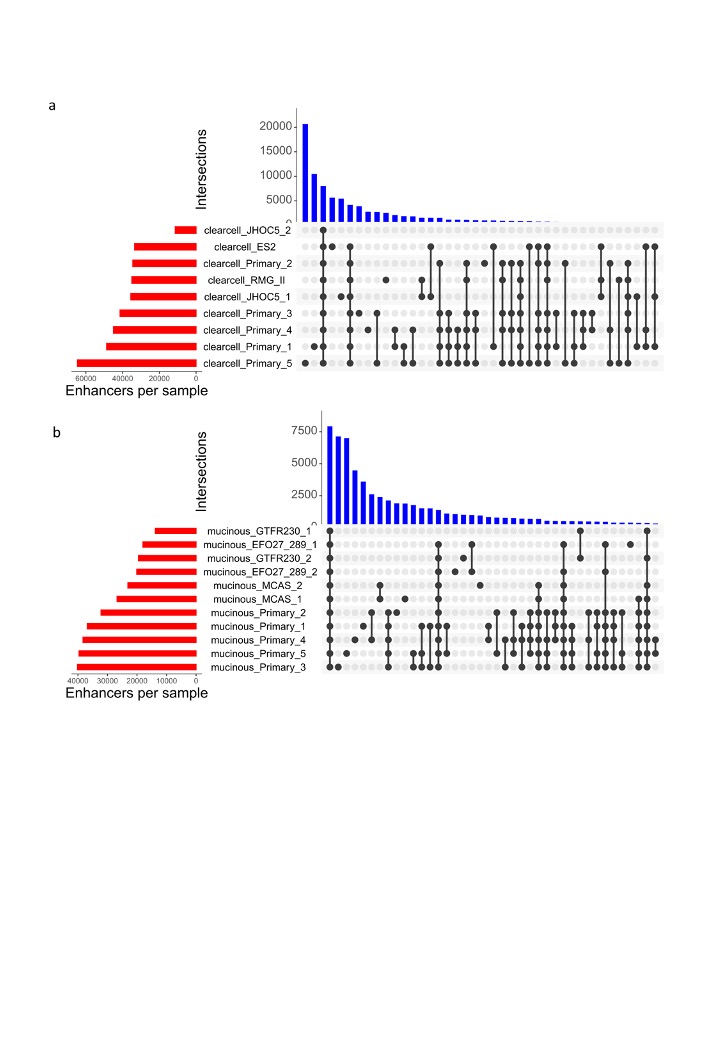


Intersections of (a) clear cell (CC) and (b) mucinous carcinoma (MC) H3K27ac occupied GeneHancer enhancer regions for all samples analysed including cell lines and primary cells.

**Supplementary Figure S2**. Coregulator distribution within enriched complexes in clear cell and mucinous carcinoma samples


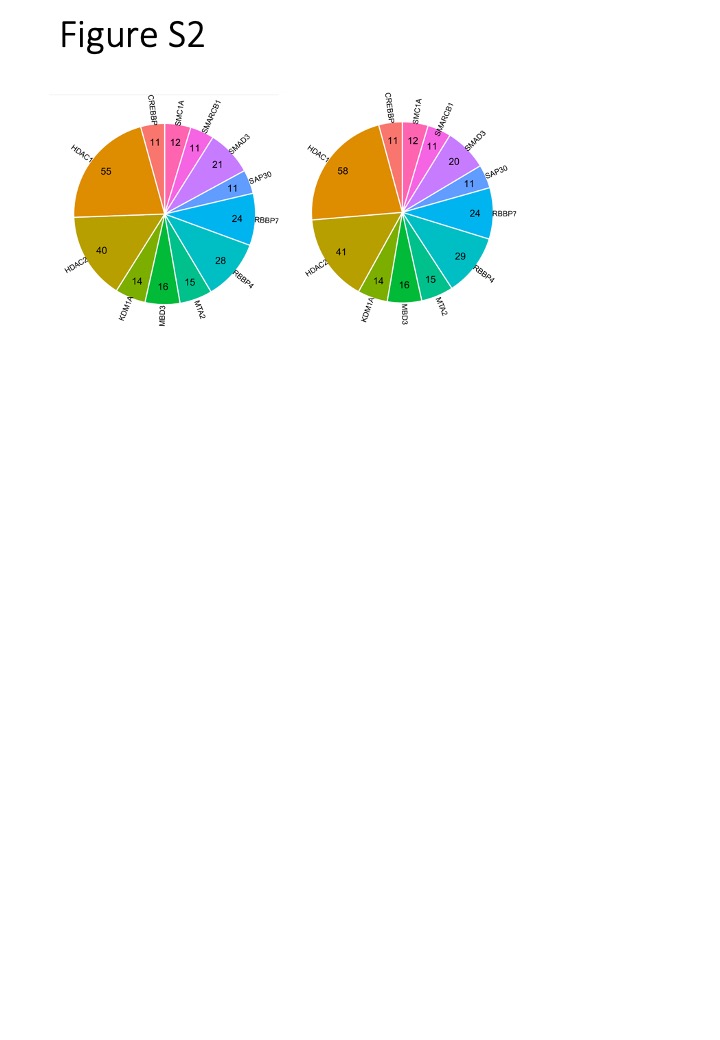


Number of complexes where each coregulator is a component for CC and MC samples respectively, including only coregulators present in more than ten complexes.

**Supplementary Figure S3.** Viability assays and IC_50_ calculations for HGSC cell lines

**
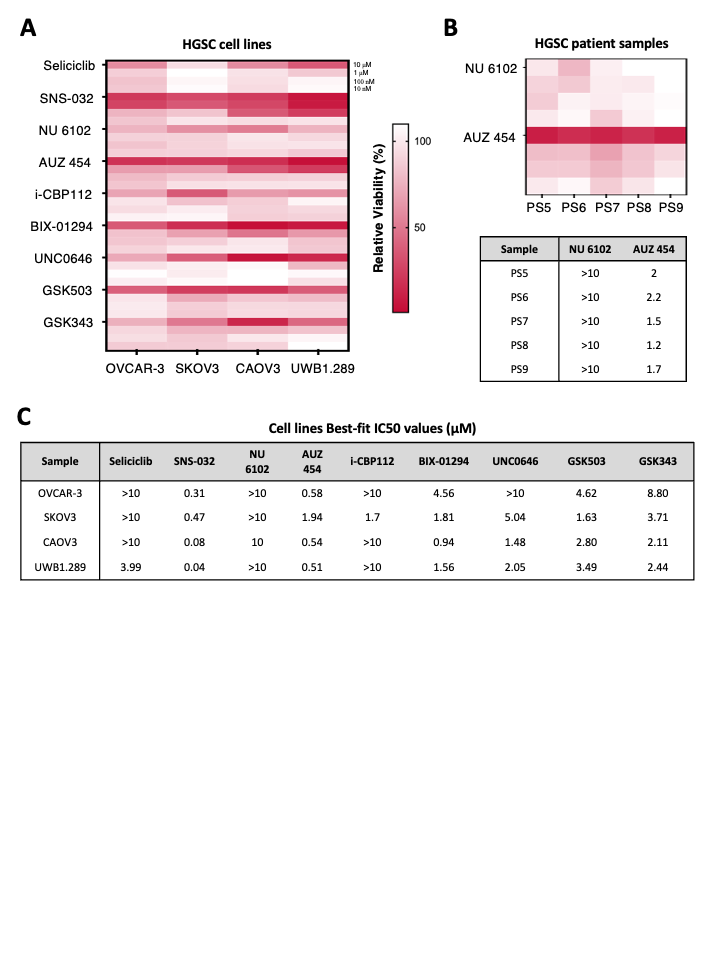
**

(A) Heatmap representing the effect of drug compounds on HGSC cell lines treated for 72h. Each 4 rows of the heatmap represent the top concentrations used to derive IC_50_ values (10μM, 1μM, 100nM and 10nM). Heatmap values were calculated using relative viability compared to the vehicle control (DMSO). Red colour indicates low viability following treatment. (B) Heatmap representing the effect of NU 6102 and AUZ 454 on patient derived samples. (C) Tables represent calculated best-fit IC50 values after 72h of treatment with compounds.

**Supplementary Figure S4.** Cell line validation of CDK2 and EHMT2 targeting

**
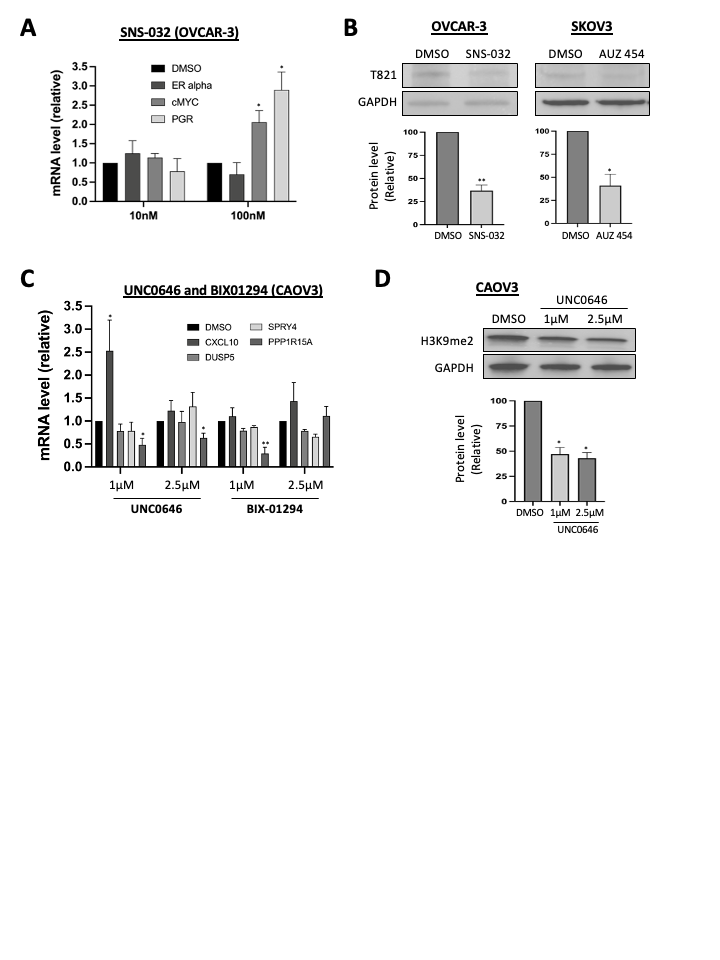
**

(A, C) Cell lysates of OVCAR-3 and CAOV3 cell lines were subjected to qRT-PCR validation to confirm changes in expression levels of ER alpha, cMYC, PGR, CXCL10, DUSP5, SPRY4 and PPP1R15A targets. (B, D) Protein lysates from OVCAR-3, SKOV3 and CAOV3 were subjected to western blot analyses to study changes in Rb phosphorylation of Threonine 821 (T821) and H3K9me2 protein levels following SNS-032 [250nM], AUZ [250nM] and UNC0646 treatment; GAPDH was used as loading control. Protein level (relative) panels display proportional differences between relative densities of T821/H3K9me2 and control samples (DMSO) calculated using ImageJ. All values represent the mean±standard deviation (SD) of three biological samples (^*^p<0.05, ^**^p<0.01).

**Supplementary Figure S5.** Flow cytometry analysis of DNA profile and apoptosis

**
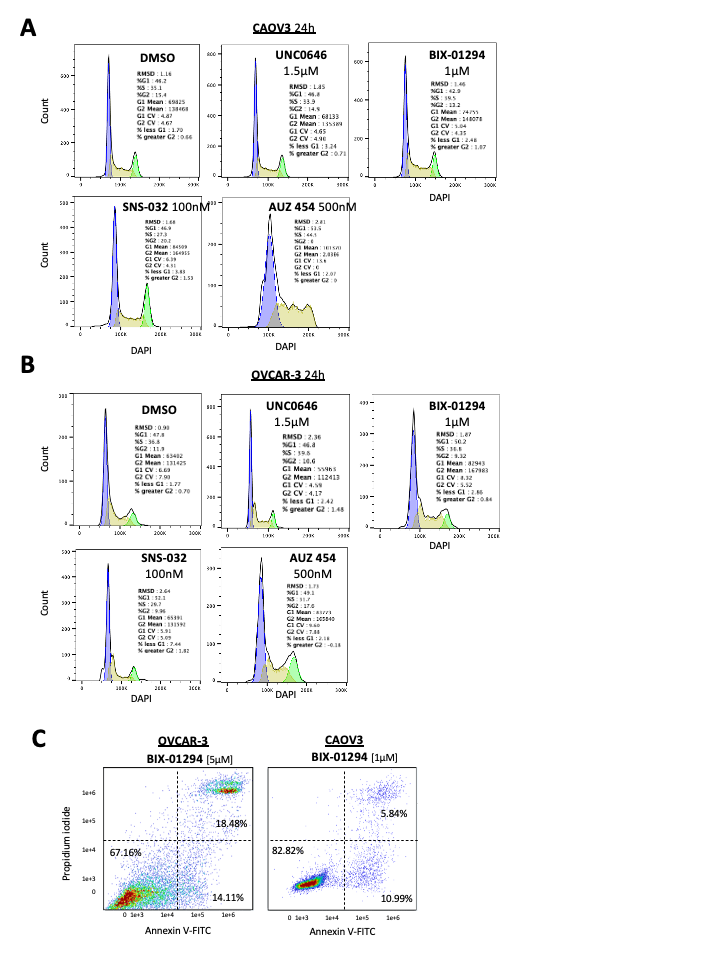
**

(A, B) Flow cytometry analysis of CAOV3 and OVCAR-3 cells treated with selected compounds for 24h. Blue peaks represent cells in G0/G1 phase, while green peaks represent cells in G2/M phase. The area depicted as yellow represents cells in S phase. (C) Flow cytometry apoptosis analysis of OVCAR-3 and CAOV3 cells treated with BIX-01294 for 24h. Cells were stained with Propidium iodide and Annexin V-FITC. Graphs display cell densities, whereby red, green and blue colours indicate high, medium and low cell densities respectively.

**Supplementary Tables**

**Supplementary Table S1**. Results of quality control analyses for downloaded ChIP-Seq datasets

**Supplementary Table S2**. List of subtype shared and specific enriched transcription factors

**Supplementary Table S3**. List of subtype shared and specific enriched complexes and coregulators

**Supplementary Table S4**. Summary of STITCH chemical-protein interaction results

**Supplementary Tables S1-4** are provided as separate excel files.

**Supplementary Table S5**. Patient-derived clinical samples utilised in this study

| **Sample** | **Age** | **Diagnosis** | **Stage** | **Chemotherapy** |
| --- | --- | --- | --- | --- |
| PS1 | 69 | HGSC | IIIC | 6 cycles |
| PS2 | 65 | HGSC | IVa | 4 cycles |
| PS3 | 59 | HGSC | IIIc | - |
| PS4 | 69 | HGSC | IIIc | - |
| PS5 | 78 | HGSC | III | 6 cycles |
| PS6 | 67 | HGSC | IIIc | 4 cycles |
| PS7 | 80 | HGSC | - | 4 cycles |
| PS8 | 71 | Serous cystadenocarcinoma | IVb | 6 cycles |
| PS9 | 60 | HGSC | IIIc | - |

**Supplementary Methods**

**RNA extraction and qRT-PCR**

Total RNA was isolated using the RNeasy Plus Mini Kit (74135, Qiagen; DE) and reverse transcribed using the high-capacity cDNA reverse transcription kit (4368814, ThermoFisher Scientific). All qRT-PCR reactions were conducted in a CFX96^™^ real-time PCR detection system (Bio-rad; CA, US) using iTaq^™^ Universal SYBR Green supermix (1725125, Bio-rad). All samples were tested in triplicates (n = 3). The experiment was replicated in the laboratory three times. When possible, synthetic oligonucleotides (sequences available upon request) span exon-exon boundaries to preclude amplification of genomic DNA. Relative gene expression was determined following the δC_t_ method [1] and normalised to an internal reference gene (RPL5). Variance was similar between the groups statistically compared. One-way ANOVA statistical analyses were performed on δC_t_ values of three biological replicates using Graphpad Prism (V9); Sidak’s test was used to correct for multiple comparisons. Graphed values represent the mean ± SD of three biological samples.

**Protein extraction and western blot**

Cells were lysed using RIPA buffer (R-0278, Merck) including 1x Halt^™^ Protease and phosphatase inhibitor cocktail (1861281, ThermoFisher Scientific). Total protein was quantified using a standardised bovine serum albumin (P06-1391050, PAN^™^-Biotech; DE). Concentration curve following the Bio-Rad DC^™^ protein assay (5000112, Bio-rad). Total protein samples were separated using SDS-PAGE gel (4568094, Bio-rad) and then transferred to PVDF membranes (1704156, Bio-rad). Primary antibody incubations were performed overnight (O/N) followed by two hours in the presence of HRP-conjugated secondary antibodies (Cytiva; anti-mouse: NA931V, anti-rabbit: NA934V). Protein intensity was detected with Clarity^™^ Western ECL substrate (Bio-rad, 170-5060). Relative protein intensity levels were calculated using ImageJ [2]. Graphical depictions of relative protein levels represent the proportional difference between a treatment and its control (100%). Variance was similar between the groups statistically compared. One-way ANOVA analyses were performed on relative intensity values using Graphpad Prism (V9); Sidak’s test was used to correct for multiple comparisons. Graphed values represent the mean ± SD of three biological samples. The following commercial antibodies were used for immune blotting: anti-GAPDH (Santa Cruz, sc-47724, AB_627678), anti-H3K9me2 (Active Motif, 39239, AB_2793199) and anti-phospho T821 (Abcam, ab4787, AB_304624).

**Supplementary References**

[1] Yuan JS, Reed A, Chen F, Stewart CN. Statistical analysis of real-time PCR data. BMC Bioinformatics 2006;7:85.

[2] Schneider CA, Rasband WS, Eliceiri KW. NIH Image to ImageJ: 25 years of image analysis. Nat Methods 2012;9:671–5.
